# From Mouse to Human: Comparative Analysis between Grey and White Matter by Synchrotron-Fourier Transformed Infrared Microspectroscopy

**DOI:** 10.3390/biom10081099

**Published:** 2020-07-24

**Authors:** Paula Sanchez-Molina, Martin Kreuzer, Núria Benseny-Cases, Tony Valente, Beatriz Almolda, Berta González, Bernardo Castellano, Alex Perálvarez-Marín

**Affiliations:** 1 Institute of Neurosciences, Universitat Autònoma de Barcelona, 08193 Bellaterra, Barcelona, Spain; paula.sanchez@uab.cat (P.S.-M.); tony.valente@uab.cat (T.V.); beatriz.almolda@uab.cat (B.A.); berta.gonzalez@uab.cat (B.G.); bernardo.castellano@uab.cat (B.C.); 2 Department of Cell Biology, Physiology and Immunology, Universitat Autònoma de Barcelona, 08193 Bellaterra, Barcelona, Spain; 3 ALBA Synchrotron Light Source, Carrer de la Llum 2-26, 08290 Cerdanyola del Vallès, Barcelona, Catalonia, Spain; mkreuzer@cells.es (M.K.); nbenseny@cells.es (N.B.-C.); 4 Biophysics Unit, Department of Biochemistry and Molecular Biology, Universitat Autònoma de Barcelona, 08193 Bellaterra, Barcelona, Catalonia, Spain

**Keywords:** infrared spectroscopy, grey matter, white matter, lipid oxidation, protein structure, central nervous system

## Abstract

Fourier Transform Infrared microspectroscopy (μFTIR) is a very useful method to analyze the biochemical properties of biological samples in situ. Many diseases affecting the central nervous system (CNS) have been studied using this method, to elucidate alterations in lipid oxidation or protein aggregation, among others. In this work, we describe in detail the characteristics between grey matter (GM) and white matter (WM) areas of the human brain by μFTIR, and we compare them with the mouse brain (strain C57BL/6), the most used animal model in neurological disorders. Our results show a clear different infrared profile between brain areas in the lipid region of both species. After applying a second derivative in the data, we established a 1.5 threshold value for the lipid/protein ratio to discriminate between GM and WM areas in non-pathological conditions. Furthermore, we demonstrated intrinsic differences of lipids and proteins by cerebral area. Lipids from GM present higher C=CH, C=O and CH_3_ functional groups compared to WM in humans and mice. Regarding proteins, GM present lower Amide II amounts and higher intramolecular β-sheet structure amounts with respect to WM in both species. However, the presence of intermolecular β-sheet structures, which is related to β-aggregation, was only observed in the GM of some human individuals. The present study defines the relevant biochemical properties of non-pathological human and mouse brains by μFTIR as a benchmark for future studies involving CNS pathological samples.

## 1. Introduction

Fourier Transform Infrared microspectroscopy (μFTIR) is an in situ method that offers the possibility of analyzing in detail the biochemical properties of fixed and non-fixed cells and tissues with no needed of staining, homogenization or further manipulations that can alter the nature of the samples. Lipid peroxidation, de/phosphorylation and protein conformations are some of the measurements that the infrared spectrum can provide with a high spatial resolution and sensitivity at the microscopic level [1,2,3].

The intrinsic chemical characteristics of a biological sample is essential information to understand pathophysiological processes. Among others, numerous studies have benefited from this method to elucidate biochemical and molecular features of different pathologies that affect the central nervous system (CNS). The brain is composed of two main areas clearly differentiated: grey matter (GM) and white matter (WM). GM is characterized by the presence of neuronal bodies, whereas WM is characterized by the absence of neuronal bodies and a high presence of myelinated axons. Depending on the disease, the affected brain area may be different and, therefore, biomedical studies are usually focused in one specific anatomical region. μFTIR analyses have been performed in different cerebral areas affected by neurodegenerative disorders, such as Alzheimer’s [4,5,6,7], Parkinson’s [8] and Huntington’s [9] diseases, to detect protein aggregation and lipid peroxidation. Infrared radiation it is also very useful to evaluate demyelinating diseases, such as multiple sclerosis, quantifying the amount and the status of lipids in WM areas. A decrease in the lipid content, together with a high lipid oxidation, is a characteristic feature observed by μFTIR in demyelinated areas [10,11,12]. In addition, some authors have studied by infrared spectroscopy the effect of CNS related pathologies on GM and WM, at the same time finding different alterations by area [9,11,13,14,15]. However, to properly understand the molecular mechanism of pathologies, the characterization of non-pathological tissues needs to be established first. Furthermore, it is important to highlight that most of the studies performed in the CNS are carried out in rodents and, in consequence, it is crucial to determine the differences and the similarities between humans and rodents with the aim of evaluating the reproducibility and applicability of this method, comparing animal models to human.

In the present study, we characterize the protein and lipid composition and their infrared spectral properties in GM and WM areas by μFTIR. Moreover, this work compares the regional-related properties observed by this method in the human brain with those of the mouse, which is the most widely used experimental model in the neuroscience field.

## 2. Materials and Methods

### 2.1. Human Samples

Postmortem human brain tissue was obtained from the Neurological Tissue Bank at the Biobanc-Hospital Clínic-IDIBAPS (Barcelona, Spain). The whole procedure was performed in accordance with the Helsinki Declaration, the Convention of the Council of Europe on Human Rights and Biomedicine and approved by the Ethical Committee of the Autonomous University of Barcelona and the Ethical Committee of Clinical Research-Hospital Clinic de Barcelona (A1-OF15016, 05/20/2015). Frontal cortex samples from individuals (*n* = 7) with no clinical neurological manifestations were used in this study (five women and two men; 78 ± 7.3 years old; 15.2 ± 6.6 h of postmortem delay).

### 2.2. Animal Samples

C57BL/6 mice (*n* = 5) were used in the present study (four females and one male). In order to mimic the experimental conditions between species, the age of the mice was 20–22 months old. Animals were maintained in conventional plastic cages with food and water ad libitum, in a 12 h light/dark cycle, at 22 ± 2 °C and 50–60% humidity. All experimental animal work was conducted according to Spanish regulations (Ley 32/2007, Real Decreto 1201/2005, Ley 9/2003 and Real Decreto 178/2004) in agreement with European Union directives (86/609/CEE, 91/628/CEE) and was approved by the Ethical Committee of the Autonomous University of Barcelona.

### 2.3. Sample Preparation

Human brains were frozen at the Neurological Tissue Bank and stored at −80 °C until use. For this study, cerebral frontal cortex samples containing grey matter (GM) and white matter (WM) areas were used. Mice were euthanized under an anesthesia solution of xylazine (30 mg/kg) and ketamine (120 mg/kg) and intracardially perfused for 10 min with 4% paraformaldehyde in 0.1 M phosphate buffer (pH 7.4). The brains were immediately post-fixed in the same solution for 4 h at 4 °C, cryoprotected with 30% sucrose solution in 0.1 M phosphate buffer for 48 h at 4 °C, frozen in ice-cold 2-methylbutane (320404, Sigma-Aldrich, St. Louis, MO, USA) and stored at −80 °C until use. The areas of study in mice were the cerebral cortex and the corpus callosum (bregma between 0.86 mm and −1.22 mm coordinates) as representative areas of GM and WM, respectively. Prior to μFTIR analysis, frozen sections from human and mouse brains containing the areas of interest were cut 8 µm thick on a cryostat (CM3050S Leica) and mounted onto polished calcium fluoride (CaF_2_) optical windows (CAFP20-1, Crystran, UK). To minimize water contribution, sections on CaF_2_ slides were air-dried at room temperature and stored in a vacuum protected from light until use.

### 2.4. μFTIR Data Acquisition

Fourier Transform Infrared microspectroscopy (μFTIR) based on synchrotron radiation was carried out at the MIRAS beamline of ALBA synchrotron light source (Catalonia, Spain) [16]. A Hyperion 3000 microscope equipped with a 36× magnification objective and coupled to a Vertex 70 spectrometer (Bruker, Billerica, MA, USA) was used. The spectra collection was performed in transmission mode at 4 cm^−1^ spectral resolution, 10 μm × 10 μm aperture dimensions and 128 scans. All spectra were obtained by means of Opus 7.5 software (Bruker, Billerica, MA, USA). The measuring range was 4000–600 cm^−1^ wavenumbers, and zero filling was performed with fast Fourier transform (FFT), so that we obtained one point every 2 cm^−1^ in the final spectra. Background spectra were collected from a clean area of each CaF_2_ window every 10 min. A mercury cadmium telluride (MCT) detector was used, and both the microscope and spectrometer were continuously purged with a flow of dried air. For each studied area, approximately 100 spectra with a step size of 50 μm × 50 μm for human samples and 30 μm × 30 μm for mouse samples were acquired. In order to represent regional differences with high spatial resolution in the tissue, maps of 200 spectra with a step size of 6 μm × 6 μm were performed in one representative sample of each species containing GM and WM areas.

### 2.5. μFTIR Spectra Analysis

Principal Component Analysis (PCA) was done using the open source software package Orange (Bioinformatics Laboratory of the University of Ljubljana, Version 3.23, Ljubljana, Slovenia) with the spectroscopy add-on (Version 0.4.9) [17]. The analysis was focused on the lipid region (3050–2800 cm^−1^) and the protein region (1800–1500 cm^−1^) of the spectra. The PCA were performed after the second derivatives were calculated and unit vector normalizations were performed on each spectrum for each region. The second derivatives were calculated with a polynomial order of 2 and a window size of 5 points for the lipid region and 17 points for the protein region. For a statistical and detailed study of lipid and protein composition, Unscrambler X software (CAMO Software, Oslo, Norway) was used. In this software, second derivation using the Savitsky–Golay algorithm with an eleven-point smoothing filter and a polynomial order of 3 was applied on the spectra to eliminate the baseline contribution and enhance narrow bands. After that, we measured the absorptions at the following wavenumbers related to functional groups of biochemical interest: ~2921 cm^−1^ (CH_2_ asymmetric stretching vibrations), ~2962 cm^−1^ (CH_3_ asymmetric stretching vibrations), ~3012 cm^−1^ (C=CH, unsaturated olefinic group), ~1743 cm^−1^ (C=O, carbonyl group), ~1656 cm^−1^ (α-helix protein structure), ~1637 cm^−1^ (intramolecular β-sheet protein structure), ~1625 cm^−1^ (intermolecular β-sheet protein structure) and ~1548 cm^−1^ (Amide II). The absorbances at the mentioned wavenumbers, 2 cm^−1^ before and at 2 cm^−1^ after, were obtained and averaged to represent the peaks of interest. CH_2_ and CH_3_, especially CH_2_, are functional groups mostly present in lipids, while α-helix and β-sheet structures belong to the Amide I functional group band present in proteins. To account for variations in the tissue thickness, CH_3_, C=CH and C=O peaks were normalized by the CH_2_ band (as an approximation of the total lipid content), whereas Amide II and β-sheet peaks were normalized by the total protein content (Amide I or α-helix bands).

### 2.6. Statistical Analysis

Statistical analysis and graphical representation were performed using the Graph Pad Prism^®^ software. To determine differences between GM and WM, Student’s *t*-test was carried out on human and mouse data. For statistically significance, we considered *p*-value < 0.05. All data are expressed as mean values ± standard error of the mean (SEM).

## 3. Results

In this section, we present the mean spectra in the range 3500–1250 cm^−1^ wavenumbers and the PCA of the lipid and protein regions of GM and WM samples belonging to humans and mice. Moreover, statistical analyses were applied for the study of CH_3_ (d^2^A_2962_/d^2^A_2921_), C=CH (d^2^A_3012_/d^2^A_2921_) and C=O (d^2^A_1743_/d^2^A_2921_) compositions with respect to the lipid content, and Amide II (d^2^A_1548_/d^2^A_1656+1637_), intramolecular β-sheet structure (d^2^A_1637_/d^2^A_1656_) and intermolecular β-sheet structure (d^2^A_1625_/d^2^A_1656+1637_) with respect to the protein content, comparing their infrared absorptions between GM and WM in human and murine brain samples.

### 3.1. Different Chemical Profile of the Brain Tissue

**Figure 1:**
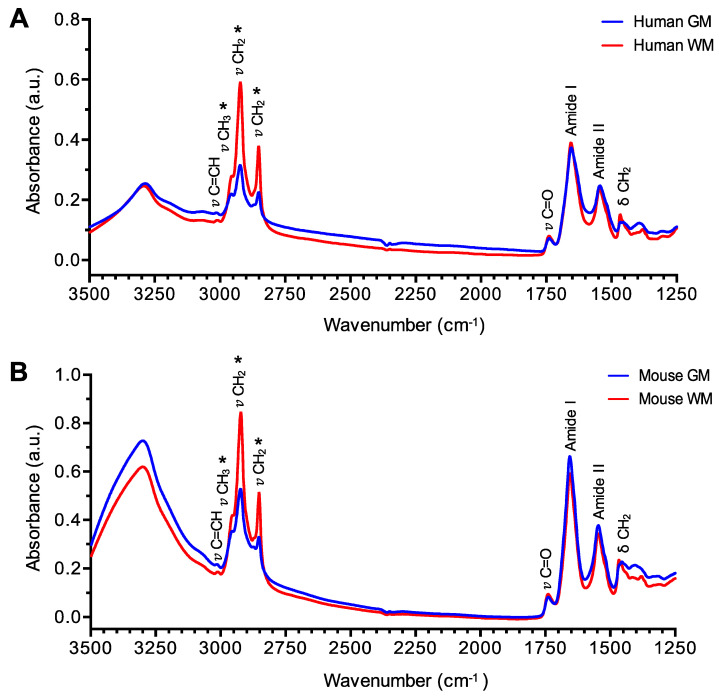
μFTIR profile of grey matter (GM) and white matter (WM). Spectra of GM (blue line) and WM (red line) areas were collected and average for human (**A**) and mouse (**B**) brain samples. Note the increased absorptions of CH_2_ and CH_3_ peaks (*) in WM with respect to GM. a.u.—arbitrary units.

**Figure 2:**
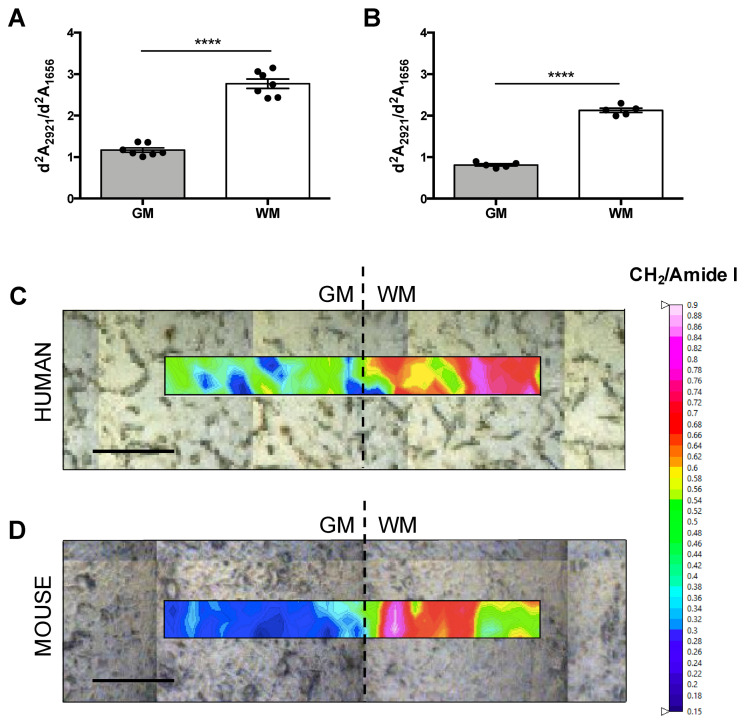
Lipid/Protein composition by μFTIR in grey matter (GM) and white matter (WM). Quantification of CH_2_/Amide I absorbance after second derivative (d^2^A_2921_/d^2^A_1656_) in humans (**A**) and mice (**B**) confirms higher lipid amount in WM with respect to GM. Each dot in the graphs correspond to the mean of approximately 100 infrared measurements from a subject. Representative in situ heat maps from human (**C**) and murine (**D**) cerebral tissue showing CH_2_/Amide I ratio. Dashed lines in the tissue images represent the limit between GM and WM. **** *p*-value < 0.0001. Scale bar = 50 μm.

In order to visualize the infrared profile of GM and WM areas, all spectra from each cerebral region were averaged in both species. μFTIR data show a clear different spectrum profile for each brain area characterized by higher CH_2_ (~2920 cm^−1^ and ~2850 cm^−1^) and CH_3_ (~2960 cm^−1^) peaks in WM with respect to the GM area; however, Amide I (~1650 cm^−1^) and Amide II (~1550 cm^−1^) peaks are very similar in both areas, as shown in Figure 1A,B. CH_2_ and CH_3_ groups are mainly related to lipids, which are very abundant in myelin, while Amide I and II groups are related to proteins. Our results demonstrate that lipid/protein (CH_2_/Amide I) amount is statistically higher in WM compared with GM, as shown in Figure 2A,B. This different chemical composition is also observed by high resolution imaging tissue maps, where GM and WM from the same sample were captured, as shown in Figure 2C,D. All the mentioned observations are present in humans and mice.

### 3.2. Principal Component Analysis of Brain Areas and Species

**Figure 3:**
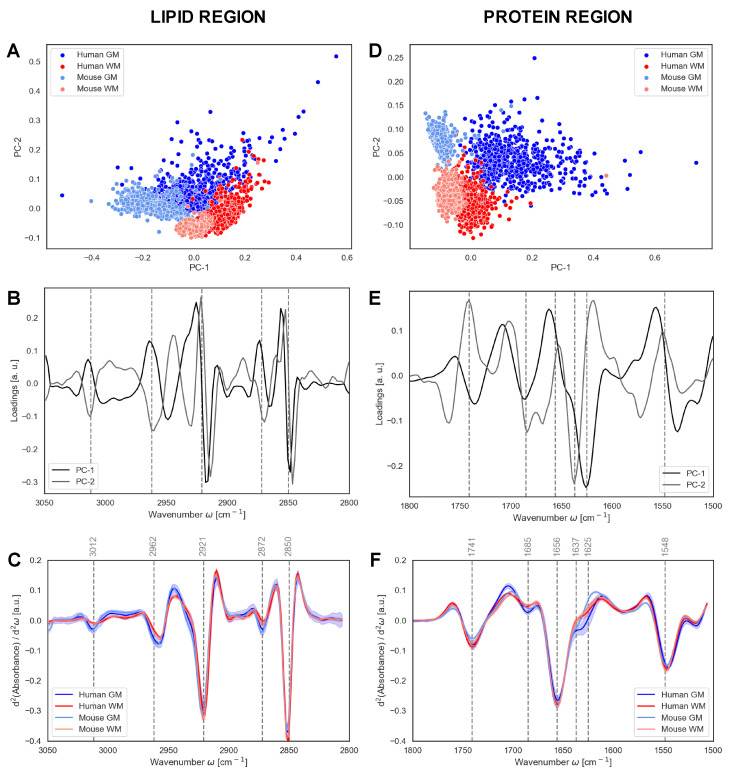
Principal Component Analysis (PCA) of grey matter (GM) and white matter (WM). PCA of the normalized second derivatives of the lipid region (3050–2800 cm^−1^) and protein region (1800–1500 cm^−1^), showing the scores plots (**A**,**D**), the resulting loadings for PC-1 and PC-2 (**B**,**E**) and the average spectra (**C**,**F**) of the two regions, respectively. Spectra shadowing in C and F indicates standard deviation. a.u.—arbitrary units.

Principal Component Analysis (PCA) was applied to calculate Euclidean distances between each spectral dataset and to cluster the spectral properties of the four sample groups (WM and GM for humans and mice). The PCA scores plots directly unravel differences and similarities among the spectra, while the PCA loadings detail the spectral differences between the clusters. The analysis is shown in Figure 3—left panels show the comparison of the C-H bond region (3050–2800 cm^−1^). This region is mainly dominated by the infrared absorption of lipids in the system. Figure 3A shows the score plot and Figure 3B shows the corresponding PC1 (59%) and PC2 (17%) loadings. The score plot distribution shows that PC1 clearly differentiates human and mouse data, while PC2 differentiates between GM and WM. PC1 loading indicates that slight shifts characterize the differences between human and mouse samples, while PC2 indicates that differences between 3012 cm^−1^ (C=CH bond), 2921 cm^−1^ (CH_2_ group) and 2962 cm^−1^ (CH_3_ group) intensities differentiate between WM and GM. For a better illustration of differences, the average spectra of the second derivative of each group are plotted in Figure 3C. Right panels in Figure 3 show the PCA corresponding to the 1800–1500 cm^−1^ region where carbonyl groups and the Amide I and II absorb. The score plot in Figure 3D shows that, as in the C–H bond region, PC1 (54%) discriminates between humans and mice and PC2 (17%) between GM and WM. The loadings in Figure 3E indicate the wavelengths that allow for distinguishing between groups. The strongest contribution arises at 1625 cm^−1^ for PC1 and at 1637 cm^−1^ for PC2, indicating more intermolecular β-sheet structures for human samples, especially in GM, and more intramolecular β-sheet structures in human and mouse GM samples. Moreover, the contribution at 1685 cm^−1^ of PC1 suggests differences in the antiparallel β-sheet structures for humans and mice. As also observed in the representation of the average spectra of the second derivative of each group, as shown in Figure 3F, small changes in protein secondary structure discriminate between human and mouse spectra. Differently, changes in the intensity of the carbonyl band with a maximum at 1741 cm^−1^ discriminate between WM and GM.

### 3.3. Protein and Lipid Properties Differ by Cerebral Region

**Figure 4:**
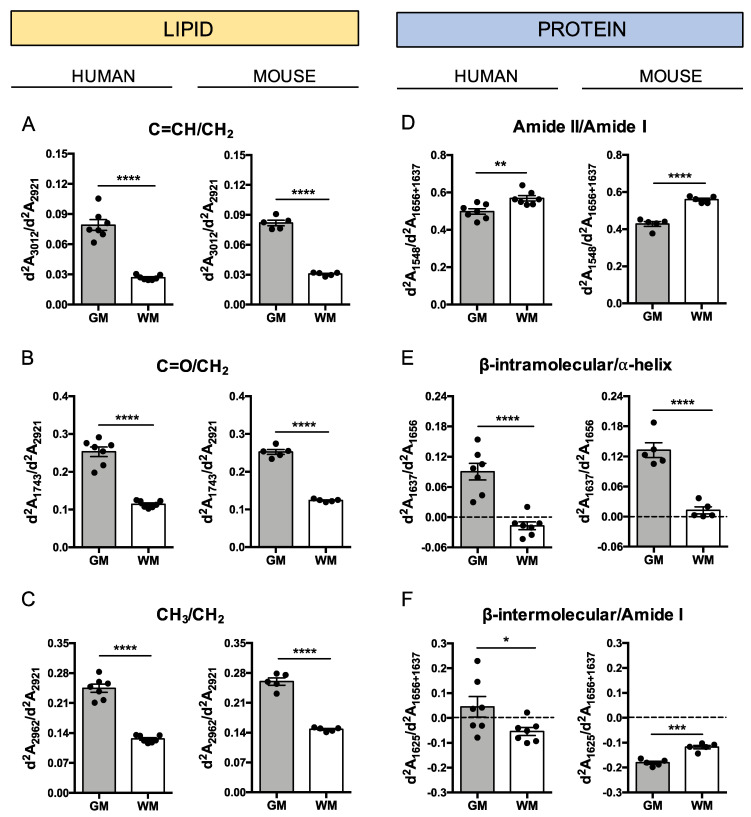
Chemical properties of lipids and proteins in grey matter (GM) and white matter (WM). Graphs represents absorbance second derivative (d^2^A) ratios of functional groups related to lipids (**A**–**C**) and proteins (**D**–**F**) in human and murine brain samples. Each dot in the graphs correspond to the mean of approximately 100 infrared measurements from a subject. * *p*-value < 0.05; ** *p*-value < 0.01; *** *p*-value < 0.001; **** *p*-value < 0.0001.

To thoroughly analyze the typical properties of lipids and proteins in brain areas, ratios for different infrared wavenumber absorptions after the second derivative were performed. For lipids, we quantified carbonyl (C=O), unsaturated olefinic (C=CH) and methyl (CH_3_) group absorbances with respect to the total lipidic amount in the sample, normalizing by the CH_2_ absorbance. Our results show that lipids in the GM area of humans and mice contain more carbonyl (d^2^A_1743_/d^2^A_2921_), olefinic (d^2^A_3012_/d^2^A_2921_) and methyl (d^2^A_2962_/d^2^A_2921_) groups with respect to the WM area, as shown in Figure 4A–C. Regarding proteins, we quantified Amide II, intramolecular β-sheet and intramolecular β-sheet absorbances with respect to the total protein amount normalizing by the Amide I absorbance. Compared to GM, our data show that proteins of WM present more absorption in the Amide II groups (d^2^A_1548_/d^2^A_1656+1637_), that account for the N–H and C–H absorption, and less intramolecular β-sheet structures (d^2^A_1637_/d^2^A_1656_) in humans and mice, as shown in Figure 4D,E. Interestingly, intermolecular β-sheet structures (d^2^A_1625_/d^2^A_1656+1637_) seem not to be present in mice, whereas in humans we can observe their presence in the GM area belonging to four of the seven subjects studied, as shown in Figure 4F. Attending to the mean values of the samples, this work also provides evidence of higher heterogeneity, especially in the C–H region, in GM than in WM, as shown in Figure 4.

## 4. Discussion

The present work shows that synchrotron-based μFTIR is a very sensitive and reproducible method for biochemical analyses of GM and WM areas of both human and mouse brain tissues. Furthermore, the molecular features reported by μFTIR based on synchrotron light can be simultaneously compared with histological features observed in the same examined sample. Depending on the tissue sample processing (e.g., paraffin embedding, freezing, chemical fixation, etc.) and the data analysis (e.g., spectral correction, normalization, derivation, etc.), the infrared values obtained may vary. Although Mazur and collaborators showed no significant differences in the infrared output of unfixed versus formalin-fixed cells [18,19], Hackett and collaborators demonstrated some slight differences in the infrared spectrum of the cryo-fixed tissue before and after formalin fixation [20]. However, our data in both cryo-fixed and paraformaldehyde-fixed tissue show a specific composition between brain areas already reported in rats [9,15,21] and humans [11], regardless of histological sample processing. A special note of caution is due for the analysis of specific wavenumbers, such as phosphate and sugars, depending on the chemistry of sample processing (phosphate buffer, sugar-based cryoprotectants, etc.). Because of differential sample processing between human and murine samples in this study, we did not analyze the phosphate region to avoid the substantial contribution of the buffer in murine samples compared to human samples.

Our infrared spectra confirm the different biochemical compositions between brain areas characterized by a higher amount of lipid content in WM compared to GM, showing a significantly higher lipid/protein (CH_2_/Amide I) ratio in WM with respect to GM. We can considerer the lipid/protein ratio to distinguish between brain areas by the μFTIR method, considering that second derivative absorption values < 1.5 correspond with measures in GM, whereas values > 1.5 correspond with measures in WM. Despite the different sample processing used between species, this discrimination can be perfectly applied to human and mouse samples. Thus, this lipid/protein 1.5 threshold value is an interesting GM versus WM discrimination factor, that may allow unsupervised μFTIR large data acquisition in human or murine whole brain samples. As a potential diagnostic method, values greater than 1.5 in GM or values lower than 1.5 in WM could be indicative of lipid and/or protein alterations in the brains of mice and humans. A similar lipid/protein threshold value (1.05) between GM and WM was established by Bonda and collaborators in rat brain cryo-fixed samples [9], supporting the use of the μFTIR method to discriminate between brain regions in humans, mice and rats. However, our data in cryo-fixed human samples show values that are slightly different to those reported in the study of Bonda et al. [9], but very similar to those of paraformaldehyde-fixed murine samples, which may, albeit unlikely, mean that mouse is more similar to human than rat as an animal model. A more likely explanation is that differential sample processing (fixation procedure) and data processing (water subtraction, calculation of area, derivation, etc.) can account for variability between studies.

In addition, we are able to clearly visualize WM and GM in the tissue by high resolution infrared maps based on lipid/protein ratio with no need of other histological or biochemical procedures. Among the many biological differences between GM and WM, one of the most significant is that GM is predominantly composed of rich-protein neuronal somas and WM by myelinated neuronal axons [22]. Since WM is mainly constituted by multilamellar myelin sheaths and the lipid composition of myelin is ~70% dry weight [23], the higher content of lipid in WM can be attributed to this multilamellar structure surrounding the axons. In addition to neurons and myelin, we have to considerer that both GM and WM are composed by glial cells [24,25]. Cellular density and intrinsic features of glial cells differ in each brain area and, therefore, they probably also participate in the specific tissue chemical composition described in this study.

The PCA allows us to identify spectral differences and similarities between the four sample groups. On the one hand, we observed that humans and mice share common spectral features (3012 cm^−1^ and 2962 cm^−1^), while the distinction can be made between GM and WM. On the other hand, strong differences for the PC1 of the protein region (1741 cm^−1^ and 1625 cm^−1^) were found between humans and mice.

Our results show that lipid characteristics vary between GM and WM in humans and mice. In both species, CH_3_/CH_2_, C=CH/CH_2_ and C=O/CH_2_ ratios were higher in GM compared to WM. Since lipids are composed mostly by long CH_2_ chains and to a lesser extent by methyl groups, CH_2_ groups are used to measure the lipid content of a sample, while CH_3_ groups could be useful to determine the lipid chain length. Cholesterol and cerebrosides are the most abundant lipids in myelin, constituting approximately 27% and 19%, respectively, of the lipids in human WM [21,26]. Whereas cholesterol is also highly present in GM, high concentration of cerebroside is specific for WM but not for GM. As an example, galactocerebrosides, the main cerebrosides present in the WM, are composed of two chains of numerous CH_2_ groups and only two CH_3_ groups at the end of the chains, arguing for a differential observation depending on lipid tail length in the ratio CH_3_/CH_2_. However, as it is shown in Benseny-Cases et al. [27], proteins have a similar number of CH_2_ and CH_3_ groups in their lateral chains, whereas lipids have significantly more CH_2_ than CH_3_ in their hydrophobic tail. Thus, changes in the lipid/protein ratio also implies changes in the C–H region (3000–2000 cm^−1^). Absorptions of olefinic C=CH functional group at 3012 cm^−1^ and C=O functional group at 1743 cm^−1^ were used to evaluate lipid unsaturation and lipid carbonyl content, respectively. C=CH and C=O bands examined by μFTIR are absent in cholesterol, galactocerebroside, sphingomyelin and sulfatide lipids, which are characteristic of WM, and present in some phospholipids [28]. Thus, our results show higher C=CH/CH_2_ and C=O/CH_2_ ratios in GM with respect to WM and are in agreement with these data. However, Krafft et al. showed a C=CH peak in galactocerebroside, sphingomyelin and sulfatide lipids by Raman spectroscopy [29]. On the other hand, it is known that free radicals mainly react with carbon double bond sites present in unsaturated lipids, resulting in carbonyl compound formation and lipid chain fragmentation [30,31,32,33]. Therefore, the high expression of C=O groups is related to the stable end-products of lipid peroxidation and, in consequence, the analyses of lipid oxidation status, measuring C=O/CH_2_ content by μFTIR, have been performed in different studies [15,27,34]. Taking these data into account, our observations suggest that the lipids in GM could be more oxidized than in WM. Since carbon double bonds are the main target of free radicals, lipid oxidation should result in less olefinic content, however, we found a higher C=CH/CH_2_ ratio in GM than WM. Some authors have attributed this high C=CH absorption to the accumulation of lipid peroxidation end-products, which contain numerous double bonds [4,35,36]. Altogether, the lower lipid content associated with the higher olefinic and carbonyl content in GM, could be interpreted as a higher oxidative status of GM with respect to WM. Brain tissue, especially WM, is very vulnerable to oxidative stress due to the presence of a rich lipid content, high oxidative metabolic activity and low antioxidant mechanisms [37]. However, in non-pathological samples, we observed more markers related to oxidation in GM than WM. The possible accumulation of oxidized lipids in GM could be due to the greater vulnerability to oxidative stress that neurons present [38].

Regarding protein composition, we also found differences between GM and WM areas. Analyzing the ratio Amide II/Amide I, we observed higher Amide II absorption (1548 cm^−1^) in human and mouse WM with respect to GM. Mathematical methods, such as deconvolution or the second derivation of the Amide I band, allow us to detect α-helix at ~1656 cm^−1^ and intramolecular β-sheet at ~1637 cm^−1^ protein secondary conformations by μFTIR [1,39,40]. Our Amide I analysis reveals that WM is composed by fewer intramolecular β-sheet structures (d^2^A_1637_/d^2^A_1656_) compared with GM. These results were observed in both human and mouse brains. In addition to the principal β-sheet structure at ~1637 cm^−1^, more β-components have been detected in the infrared spectra after resolution enhancement. The presence of a band at ~1625 cm^−1^ represents an unusual intermolecular β-sheet structure [41] that has been observed in β-amyloid plaques [4,42,43,44], α-synuclein [45] and prion proteins [46]. In consequence, this band is used as a marker of protein β-aggregation in several pathologies [5,6,9,45,46,47]. Infrared absorption intensity of the ratio d^2^A_1625_/d^2^A_1656+1637_ after second derivative shows negative values in human WM and in both mouse brain areas. This low vibrational intensity at 1625 cm^−1^ could be due to no expression of aggregated proteins in these areas. Interestingly, some humans show positive values at this wavelength in GM. Considering studied samples belong to aged humans, the apparition of this peak could be due to the age-dependent onset of protein aggregation [48,49,50]. Murine samples also belong to aged mice and there is no expression of this band. This finding may indicate that in mice there is no production of β-aggregation in non-pathological conditions, while humans are susceptible to produce it in GM. Furthermore, a weak band at ~1685 cm^−1^, which is assigned to antiparallel β-sheet structures [41,51], was also observed in the GM of humans. The antiparallel β-sheet structure has been described as a signature of β-amyloid oligomers being absent in fibrils [42,52]. Thereby, infrared absorptions at both 1685 cm^−1^ and 1625 cm^−1^ in the GM of some individuals could suggest the presence of oligomeric β-amyloid aggregates. This assumption is consistent with the fact that only genetically modified mice are capable to accumulate β-amyloid [53], and on the contrary, has been reported that healthy elder humans can present β-amyloid plaques in GM areas [48,49,50]. Furthermore, the β-aggregation, observed specifically in the GM of some individuals, is in agreement with the weaker mechanisms that present neurons to avoid protein aggregation with respect to other cells types of the CNS [54,55]. Nevertheless, β-aggregation in healthy aged individuals and in pathophysiology, both in human and murine samples, raises an issue that requires further research.

In summary, this work demonstrates by μFTIR: (i) a specific lipid/protein composition of GM and WM areas; (ii) a different presence of lipid and protein functional groups by cerebral area; (iii) that the regional differences observed in the human brain are fairly reproduced in mice, with the exception of β-aggregation content. These similarities between species in non-pathological conditions make the mouse a good experimental animal model. µFTIR comparative biology studies, but also comparative sample and data processing studies [18,19,20] are essential to assess and understand differences and similarities between species, especially for disease-related animal models. Specifically, the study of lipid-related disorders in mice could be very reliable in humans due to the high reproducibility observed in the lipid characteristics of GM and WM; however, the study of protein-related pathologies in mouse models requires a proper assessment sample and data processing variables.

## 5. Conclusions

The understanding of the brain biochemical properties in non-pathological conditions shown in this work will help us to study the molecular dynamics in different pathologies affecting GM, WM or both. Interestingly, this study provides evidence of mice (strain C57BL/6) as good animal models to reproduce human properties of the brain. Thus, μFTIR is a powerful tool to progress in the knowledge of CNS physiology and pathology performed in mice, by which data can be extrapolated to humans.
